# Association of acute myocardial infarction with influenza: A nationwide observational study

**DOI:** 10.1371/journal.pone.0236866

**Published:** 2020-08-06

**Authors:** Moman A. Mohammad, Johan Tham, Sasha Koul, Rebecca Rylance, Cecilia Bergh, David Erlinge, Ole Fröbert

**Affiliations:** 1 Department of Cardiology, Clinical Sciences Lund University, Lund, Sweden; 2 Infectious Diseases Unit, Department of Clinical Sciences, Lund University, Malmö, Sweden; 3 Clinical Epidemiology and Biostatistics, School of Medical Sciences, Örebro University, Örebro, Sweden; 4 Department of Cardiology, Faculty of Medicine and Health; Örebro University, Örebro, Sweden; Erasmus Medical Center, NETHERLANDS

## Abstract

**Introduction:**

Influenza may precipitate cardiovascular disease, but influenza typically peaks in winter, coinciding with other triggers of myocardial infarction (MI) such as low air temperature, high wind velocity, low atmospheric pressure, and short sunshine duration.

**Objective:**

We aimed to determine the relationship of week-to-week variation in influenza cases and acute MI, controlling for meteorological factors in a nationwide population.

**Methods:**

Weekly laboratory-confirmed influenza case reports were obtained from the Public Health Agency of Sweden from 2009 to 2016 and merged with the nationwide SWEDEHEART MI registry. Weekly incidence of MI was studied with regard to number of influenza cases stratified into tertiles of 0–16, 17–164, and >164 cases/week. Incidence rate ratios (IRR) were calculated using a count regression model for each category and compared to a non-influenza period as reference, controlling for air temperature, atmospheric pressure, wind velocity, and sunshine duration.

**Results:**

A total of 133562 MI events was reported to the registry during the study period. Weeks with influenza cases were associated with higher incidence of MI than those without in unadjusted analysis for overall MI, ST-elevation MI and non ST-elevation MI independently. During the influenza season, weeks with 0–16 reported cases/week were not associated with MI incidence after adjusting for weather parameters, adjusted IRR for MI was 1.03 (95% CI 1.00–1.06, *P* = 0.09). However, weeks with more cases reported were associated with MI incidence: 17–163 reported cases/week, adjusted IRR = 1.05 (95% CI 1.02–1.08, *P* = 0.003); and for ≥164 cases/week, the IRR = 1.06 (95% CI 1.02–1.09, *P* = 0.002). Results were consistent across a large range of subgroups.

**Conclusions:**

In this nationwide observational study, we found an association of incidence of MI with incidence of influenza cases beyond what could be explained by meteorological factors.

## Introduction

Incidence of acute coronary syndrome (ACS) shows circadian, circaseptan, and circannual variation [1, 2]. Increased incidence of influenza during autumn and winter may contribute to this variation. Covariation between influenza and cardiovascular disease was described in influenza epidemics from 1915 to 1929, including the 1918–1920 pandemic, and observational studies have subsequently documented similar associations [3]. In two case series studies of more than 33 000 patients, the risk of acute myocardial infarction (MI) during 3–7 days following medical contact for acute respiratory infection was significantly increased [4, 5]. A meta-analysis of 6,735 patients with ACS and patients at risk for cardiovascular disease, both from randomized trials, found that influenza vaccination was associated with a lower risk of cardiovascular events (2.9% vs. 4.7%, risk ratio 0.64, 95% CI, 0.48–0.86) [6].

Associations of influenza with cardiovascular disease burden have been investigated with ecological, case-control, and case-only studies, as well as cohort studies, with differing results [4]. However, few studies have controlled for the fact that influenza typically peaks in winter, and weather could partly account for increased cardiovascular event incidence [7]. The goal of this study was to determine the week-to-week variation in incidence of MI and fatal MI relative to influenza incidence in a nationwide population using the Swedish Web-system for Enhancement and Development of Evidence-based care in Heart disease Evaluated According to Recommended Therapies (SWEDEHEART) registry, after controlling for key meteorological parameters as potential confounders.

## Materials and methods

### National registries

The SWEDEHEART registry provides data from Sweden with continuous registration of all deaths and percutaneous coronary interventions with a high level of completeness [8–10]. All cases of MI admitted to any coronary care unit reported to SWEDEHEART were included in the study. Information on background characteristics such as age, body mass index, smoking status, and electrocardiographic findings as well as other examinations, interventions, complications, discharge medications, and diagnoses are collected in the SWEDEHEART registry. MI diagnosis was set based by the treating physician’s assessment of patient at discharge according to the fourth universal definition of MI. Data on deaths were obtained from the National Population Registry. Weekly influenza reports were obtained from the Public Health Agency (PHA), which monitors the epidemiology of influenza through a number of surveillance systems including laboratory reports with confirmed positive PCR test data. The number of laboratory-confirmed influenza cases reported for each of the 21 counties in Sweden was used in the study.

### Statement of ethics

The Regional Ethical Review Board of Lund approved this study. The SWEDEHEART registry is an anonymized quality registry and patients are informed about their participation and their right to decline participation. Therefore, no informed consent is legally required for patient inclusion.

### Study design and population

Weekly incidence of laboratory-confirmed influenza cases from the PHA registry were merged with the SWEDEHEART registry for week of MI symptom onset. The ISO 8601 standard for calendar weeks was used. The study period was set from calendar week one of 2009 through calendar week 40 of 2016. An arbitrary and prespecified categorization of number of laboratory-confirmed influenza cases into tertiles of 0–16, 17–163, and >164 cases of influenza/week was done to aid interpretation of results. The reference period was calendar weeks 21–39, considered an influenza free season. An exception to this was done with regard to influenza season 2009/2010 which started calendar week 20 2009 and continued until calendar week 20 2010 [11]. The influenza free period was calendar week 1–19 and thus comprised the reference period for this season. Daily weather data available online from the Swedish Meteorological and Hydrological Institute website (www.smhi.se) were downloaded for each city in Sweden with a coronary care unit. The nationwide average minimum air temperature, wind velocity, sunshine duration, and atmospheric pressure for each week in the defined study period were calculated and included in the multivariable analyses. The primary endpoint was incident acute MI and fatal MI defined as MI with subsequent death within 30 days due to any cause. Secondary endpoints were incident MI categorized as ST-elevation MI (STEMI) and non-ST-elevation MI (NSTEMI). The Regional Ethical Review Board of Lund approved this study.

### Statistical analyses

Continuous variables are presented as mean ± standard deviation and categorical variables as counts and percentages. Differences in continuous parametric variables were assessed by analysis of variance. Differences in categorical variables were analysed with Chi-square test. A Poisson regression model was fitted as the primary statistical model to calculate incidence rate ratios (IRR) for each tertile relative to the reference period. The model calculated the incidence rate of MI within each tertile of influenza burden and divided these rates by the incidence rate in the reference period. Zero inflation in counts of MI was not present, but due to overdispersion, a negative binomial regression model was used instead of the Poisson. Because incidence of MI showed seasonal variation with higher incidence during winter and periods of cold temperatures, a multivariable model was applied to control for weather confounders. Adjustments were made for mean air temperature, atmospheric pressure, wind velocity, and sunshine duration. Predefined subgroup analyses were conducted using information collected at the time of the incident MI. The subgroups comprised male vs. female, age ≥75 years vs. age <75 years, presence vs. absence of known diabetes, hypertension, and coronary artery disease (CAD) as well as with vs. without therapy with beta-blockers, calcium-inhibitors, aspirin, angiotensin converting enzyme inhibitor/angiotensin receptor blocker (ACE-I/ARB), statins, and diuretics. A separate analysis of patients with MI not surviving 30 days due to any cause was conducted. A sensitivity analysis adjusting for month was performed to control for potential season-dependent effects. A sensitivity analysis of influenza season only was also conducted, excluding calendar week 21–39. In this analysis, weeks with lowest rates of influenza (0–16 cases of influenza per week) were set as the reference period instead. Finally, we conducted an analysis excluding influenza season 2009/2010 due to the longer season with a higher peak in influenza cases. All statistical analyses were performed using STATA version 14.1 for Macintosh, StataCorp, Texas. A two-sided *P*<0.05 was considered significant.

## Results

### Patient characteristics

A total of 133 562 patients with MI were reported in the SWEDEHEART registry during the study period, of which 44 055 experienced STEMI. Background demographics of patients in time periods differing in incidence of influenza showed only minor differences (Table 1). The group of patients admitted for MI during peak influenza periods (≥164 cases/week) showed significantly higher rates of previous CAD (33.1% vs. 32.0% in the reference period), hypertension, and beta-blocker and statin therapy prior to admission.

**Table 1:** Patient characteristics stratified by number of influenza cases/week.

Patient characteristics	Reference period	0–16 influenza cases/week	17–163 influenza cases/week	≥164 influenza cases/week	P-value
	n = 48,457	n = 28,292	n = 28,138	n = 28,675	
Influenza cases (median)		6 (2–9)	64 (33–100)	402 (266–658)	
STEMI/LBBB	15,947 (32.9%)	9,442 (33.4%)	9,335 (33.2%)	9,331 (32.5%)	0.17
NSTEMI	32,510 (67.1%)	18,850 (66.6%)	18,803 (66.8%)	19,344 (67.5%)	
Men	31,549 (65.1%)	18,443 (65.2%)	18,341 (65.2%)	18,643 (65.0%)	0.97
Women	16,908 (34.9%)	9,849 (34.8%)	9,797 (34.8%)	10,032 (35.0%)	
Age mean±sd	71±12	71±12	71±12	71±12	0.62
Age<75 years	27,465 (56.7%)	15,929 (56.3%)	15,923 (56.6%)	16,323 (56.9%)	0.51
Age≥75 years	20,992 (43.3%)	12,363 (43.7%)	12,215 (43.4%)	12,352 (43.1%)	
BMI median (IQR)	26 (24–29)	26 (24–29)	26 (24–29)	26 (24–29)	0.010
Current smoker	9,713 (20.0%)	5,621 (19.9%)	5,553 (19.7%)	5,544 (19.3%)	0.12
Past medical history					
Diabetes	11,240 (23.2%)	6,471 (22.9%)	6,618 (23.5%)	6,805 (23.7%)	0.074
Hypertension	25,131 (51.9%)	14,484 (51.2%)	14,505 (51.5%)	15,076 (52.6%)	0.008
CAD	15,528 (32.0%)	9,230 (32.6%)	9,216 (32.8%)	9,479 (33.1%)	0.023
Medications					
Beta-blockers	19,756 (40.8%)	11,713 (41.4%)	11,714 (41.6%)	11,930 (41.6%)	0.046
Ca-inhibitors	9,924 (20.5%)	5,841 (20.6%)	5,762 (20.5%)	5,885 (20.5%)	0.95
Aspirin	18,955 (39.1%)	11,482 (40.6%)	11,395 (40.5%)	11,423 (39.8%)	<0.001
ACE-I/ARB	19,159 (39.5%)	11,211 (39.6%)	11,173 (39.7%)	11,627 (40.5%)	0.036
Statins	16,157 (33.3%)	9,487 (33.5%)	9,601 (34.1%)	9,845 (34.3%)	0.016
Diuretics	12,463 (25.7%)	7,383 (26.1%)	7,342 (26.1%)	7,374 (25.7%)	0.49
In-hospital characteristics					
Angiography	37,424 (77.2%)	21,756 (76.9%)	21,613 (76.8%)	22,157 (77.3%)	0.41
PCI	29,660 (61.2%)	17,092 (60.4%)	17,231 (61.2%)	17,724 (61.8%)	0.008
Discharge medications					
Beta-blockers	41,406 (86.4%)	24,499 (87.0%)	24,311 (86.9%)	24,451 (86.5%)	0.036
Ca-inhibitors	8,788 (18.3%)	5,198 (18.5%)	5,172 (18.5%)	5,244 (18.5%)	0.62
Aspirin	43,180 (90.1%)	25,487 (90.5%)	25,255 (90.3%)	25,363 (89.7%)	0.011
ACE-I/ARB	36,181 (74.7%)	21,226 (75.0%)	21,229 (75.4%)	21,664 (75.6%)	0.020
Statins	40,961 (85.4%)	23,925 (85.0%)	23,905 (85.5%)	24,283 (85.9%)	0.015
Diuretics	14,555 (30.4%)	8,779 (31.2%)	8,795 (31.5%)	8,590 (30.4%)	0.006
Influenza season					
2009–2010		7,533 (26.6%)	5,445 (19.4%)	3,268 (11.4%)	<0.001
2010–2011		4,126 (14.6%)	3,257 (11.6%)	3,210 (11.2%)	
2011–2012		4,036 (14.3%)	4,637 (16.5%)	2,680 (9.3%)	
2012–2013		3,092 (10.9%)	2,776 (9.9%)	5,820 (20.3%)	
2013–2014		3,922 (13.9%)	6,169 (21.9%)	1,847 (6.4%)	
2014–2015		3,471 (12.3%)	2,024 (7.2%)	5,962 (20.8%)	
2015–2016		2,112 (7.5%)	3,830 (13.6%)	5,888 (20.5%)	

ACE-I/ARB = angiotensin converting enzyme inhibitor/angiotensin receptor blocker; CAD = coronary artery disease.

### Influenza seasons

**Fig 1:**
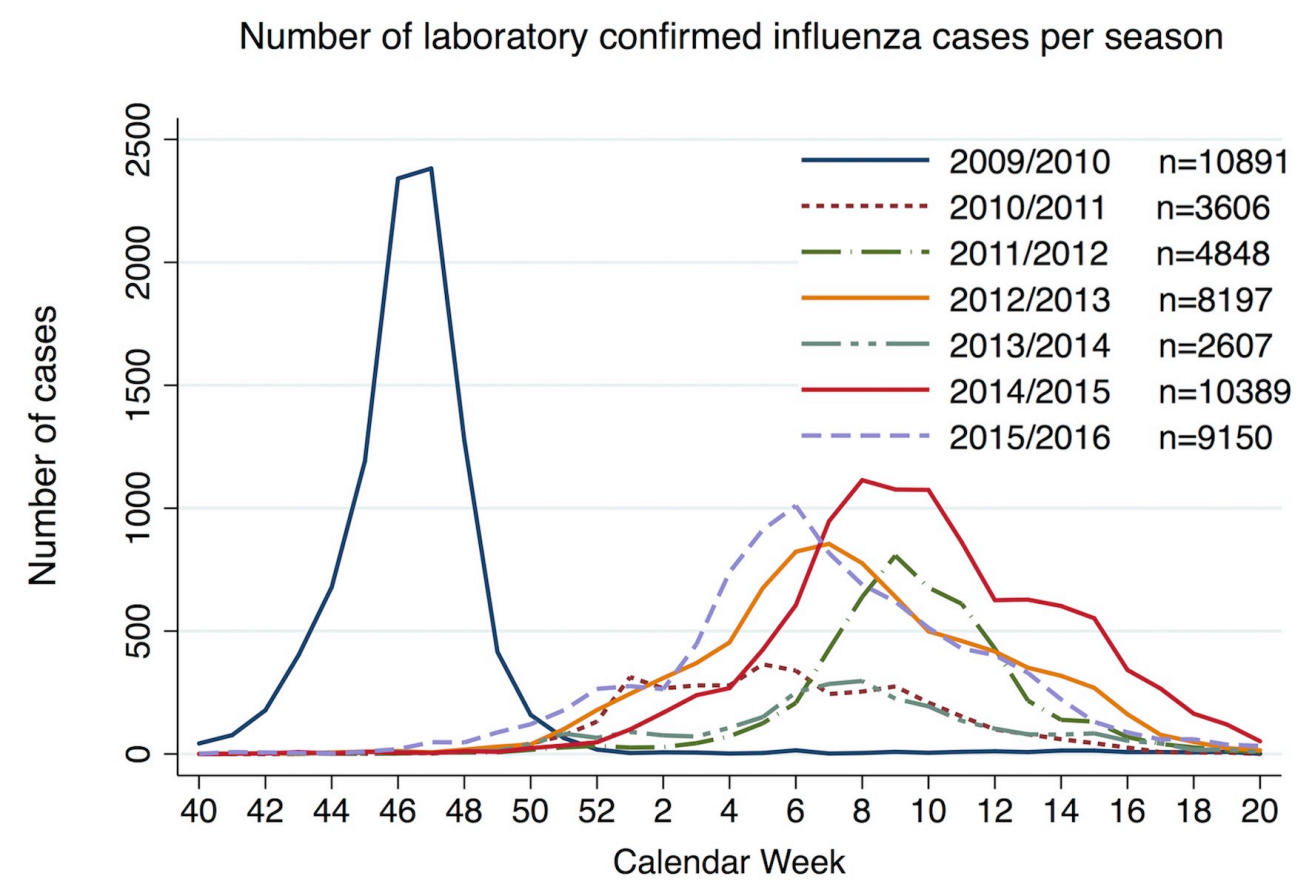
Number of influenza cases per week relative to influenza season. Number of influenza cases per week for each season. Calendar week 53 for the influenza season 2009/2010 and 2015/2016 were omitted. Fourteen influenza cases were reported for calendar week 53 in 2009 and 272 cases in calendar week 53 in 2015.

A significant difference in rates of influenza cases reported to PHA was observed among the years studied. The highest rates were observed in the 2009/2010 season with 10 891 reported cases, followed by 2014/2015with 10 389 cases (Fig 1). The season with lowest rates of laboratory-diagnosed influenza cases was 2013/2014 with 2607 cases reported.

### Primary endpoints

Weeks with influenza cases reported to PHA were associated with a significantly higher incidence of MI. During weeks with 0–16 influenza cases reported, the unadjusted incidence rate ratio (IRR) for MI was 1.04 (95% confidence interval [CI] 1.01–1.07, *P* = 0.007). Weeks with 17–163 reported cases, the incidence of MI was IRR = 1.07 (95% CI 1.04–1.10, *P*≤0.001), and, in weeks with ≥164 cases, the incidence of MI was IRR = 1.08 (95% CI 1.05–1.11, *P*≤0.001) compared to the non-flu period. Similar results were observed after adjusting for weather parameters except for the tertile with lowest reports of influenza cases: 0–16 cases/week adjusted IRR for MI was 1.03 (95% CI 1.00–1.06, *P* = 0.09). Weeks with were associated with MI incidence, adjusted IRR = 1.05 (95% CI 1.02–1.08, *P* = 0.003); as well as weeks with ≥164 cases reported, IRR = 1.06 (95% CI 1.02–1.09, *P* = 0.002).

### Secondary endpoints

**Fig 2:**
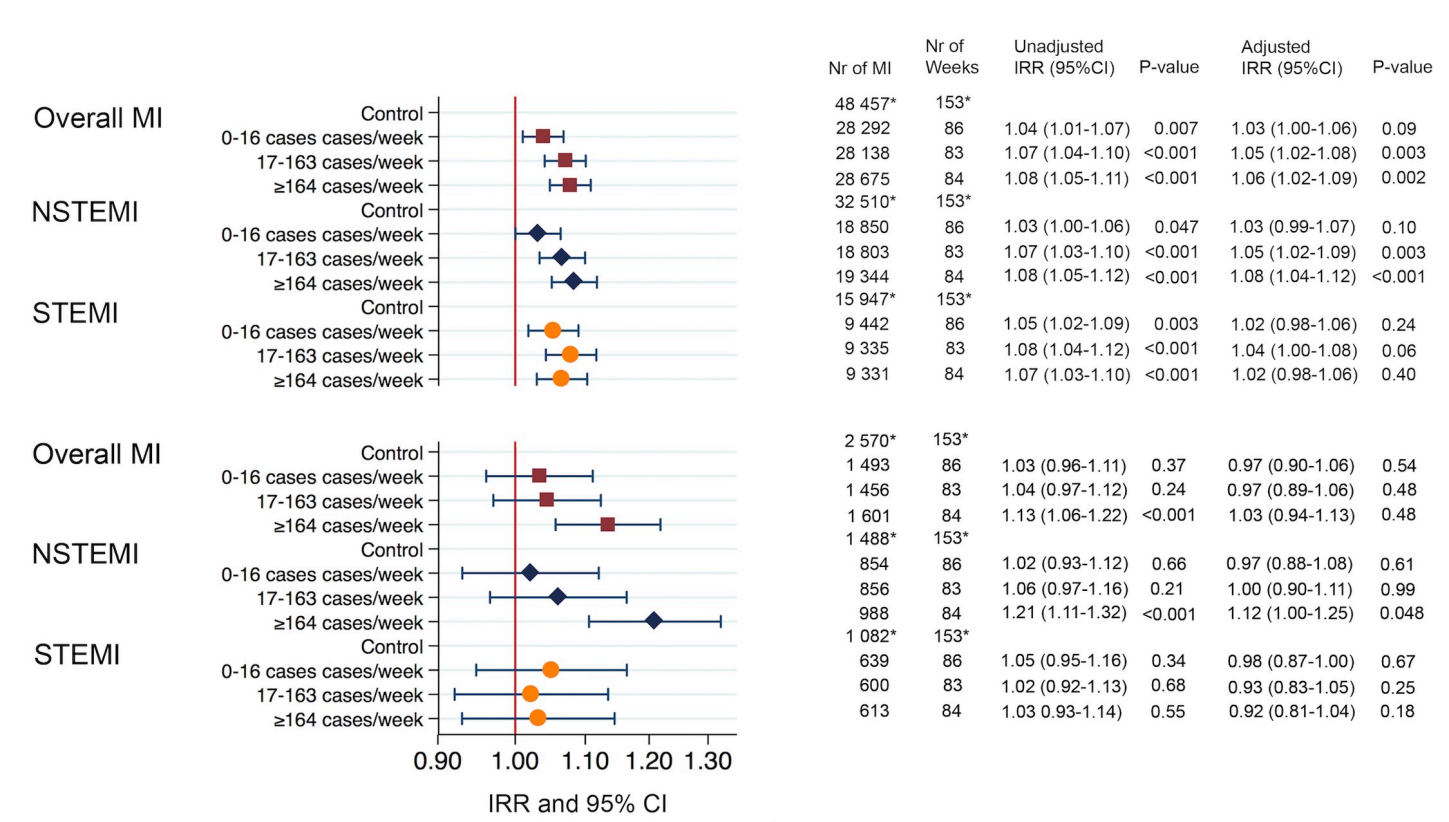
Unadjusted and adjusted Incidence Rate Ratios (IRR) of MI and fatal MI within 30 days. Fatal MI was defined as MI with subsequent death due to any cause, taking into consideration bias due to loss of follow-up. The multivariable analysis was controlled for air temperature, wind velocity, atmospheric pressure, and sunshine duration. * = The reference period was calendar weeks 21–39 identified as a period with no influenza activity.

Results of secondary endpoints were consistent with those of the primary, with significantly higher incidence of non-fatal STEMI and NSTEMI in weeks with high rates of influenza compared to the reference period (Fig 2). After adjustment for meteorological factors, higher incidence of MI was observed in weeks with more than 17 cases of influenza, driven by a higher incidence of NSTEMI. The associated risk increase of STEMI was not significant after adjustment for weather (Fig 2). In addition to a higher incidence of MI during weeks with high incidence of influenza, influenza was associated with significantly greater incidence of fatal MI in the unadjusted model, IRR = 1.13 (95% CI 1.06–1.22, *P* = <0.001), but this association was not significant after controlling for weather. Influenza was associated with higher incidence of fatal NSTEMI in unadjusted and adjusted models. No association of fatal MI in STEMI and influenza was observed.

### Subgroup analyses

**Fig 3:**
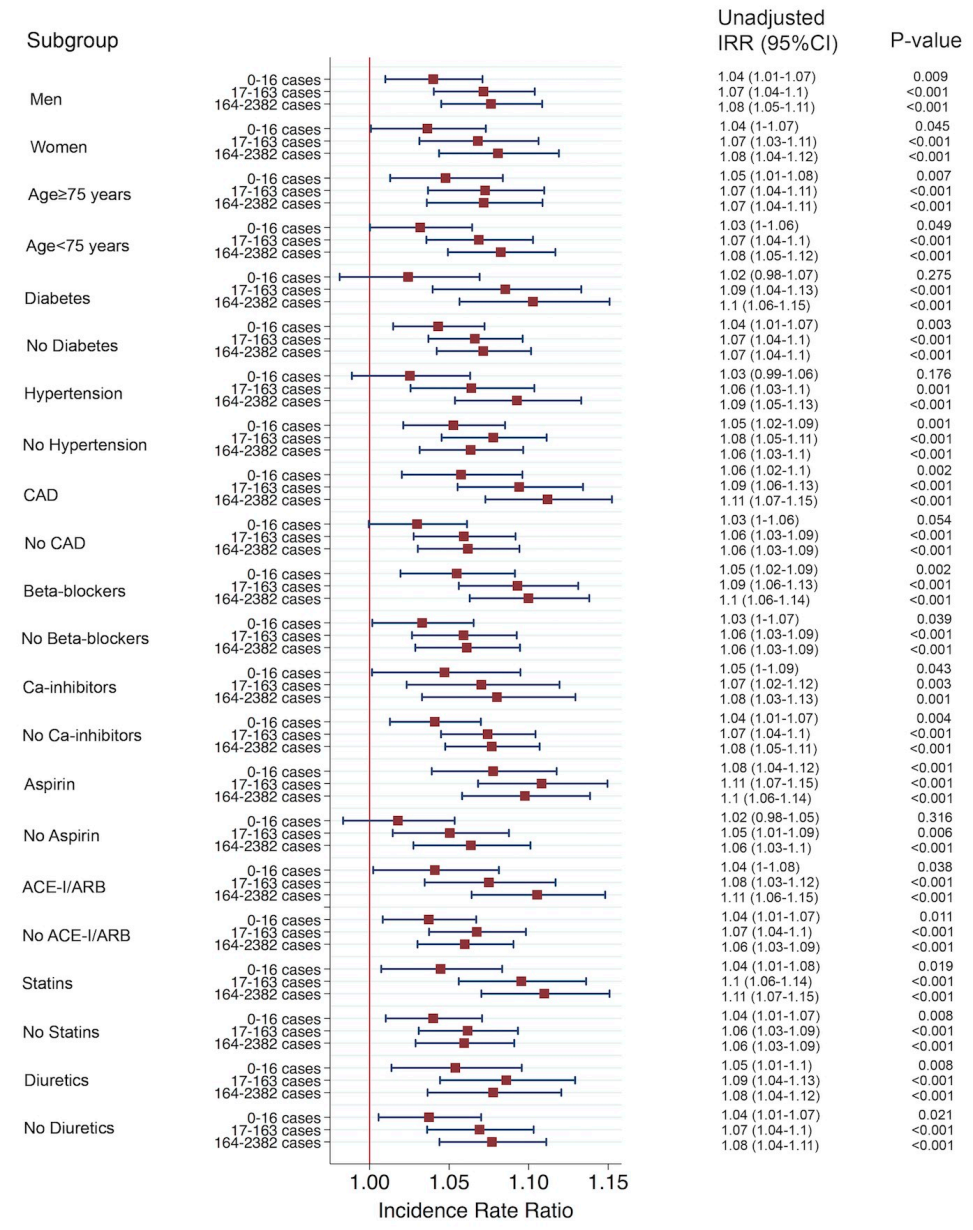
Subgroup analyses. Unadjusted incidence rate ratio (IRR) with 95% confidence interval for each subgroup. The reference period was a period with no influenza activity identified as calendar week 21–39. ACE-I/ARB = angiotensin converting enzyme inhibitor/angiotensin receptor blocker; CAD = coronary artery disease. Subgroup analyses of fatal MIs are presented in S1 Table.

Results consistently showed a significantly higher incidence of MI in periods with higher numbers of reported influenza cases across most subgroups (Fig 3).

### Sensitivity analyses

After adjustment for month, the association of influenza rate with risk of MI was attenuated but the associated risk of NSTEMI remained consistent. The sensitivity analyses of influenza seasons showed greater risk of MI and NSTEMI separately during weeks with higher rates of influenza cases (S1 Table). Excluding the influenza season 2009/2010 showed consistent associations of higher incidence of MI on weeks with higher rates of influenza reports (S1 Table).

## Discussion

In this large nationwide study, we found significantly higher occurrence of MI during weeks with influenza, increasing with influenza burden. Influenza incidence was positively associated with risk of fatal NSTEMI, but no association of mortality in STEMI and influenza was observed.

In previous studies, it has been hypothesized that the influenza virus takes opportunistic advantage of seasonal environmental factors, especially those related to weather, and that such factors are primarily to blame for increased MI incidence [12, 13]. In a recent study, we demonstrated that low temperature, low atmospheric pressure, reduced sunshine hours, and strong winds, factors often not considered in studies of influenza and MI, are all related to MI incidence. In the present study we adjusted for meteorological factors and found that the association between influenza occurrence and MI prevailed. The increase in MI with increase in influenza cases is indicative of influenza as a trigger of cardiovascular events.

A previous self-controlled case series analysis found that MI risk was higher after consultation for acute respiratory infection, most pronounced in individuals ≥80 years of age [14]. In our study, the risk for patients younger and older than 75 years was similar (Fig 3), but, because a link between individual MI and influenza cases was not possible with our design, findings may be susceptible to confounding.

Accumulating research suggests influenza as a possible MI trigger. Ecological, case-control, case-only, and cohort studies as well as small randomized trials on influenza vaccine support an influenza-MI association and point to a causal link [4].

The pathophysiological background for influenza as an MI trigger includes cytokine production leading to plaque destabilization and rupture, triggering the coagulation cascade [15–17]. However, the jury is still out on whether influenza vaccination protects against ACS. Three randomized controlled trials, underpowered for hard clinical endpoints, suggest that this might be the case [18–20]. Despite lack of high quality clinical trial data influenza vaccination for patients with coronary artery disease is recommended by the American Heart Association and the American College of Cardiology (Class I, level of evidence B recommendation) and ‘may be considered’ according to The European Society of Cardiology (Class IIb, level of evidence C) [21, 22]. However, a Cochrane review of influenza vaccination in cardiovascular disease concluded that additional higher-quality evidence is necessary to confirm whether influenza vaccination is effective in preventing cardiovascular events [23] Two large randomised clinical trials are ongoing, one placebo-controlled and one comparing high dose influenza vaccine with the standard dose [24, 25].

In this study, we found that, after controlling for weather parameters, there was not a significantly higher risk for STEMI with increase in influenza cases, while a positive association between NSTEMI and influenza persisted. In our recent study of weather and MI, lower temperatures were associated with higher incidence rates of both STEMI and NSTEMI, but the association across subgroups and sensitivity analyses were stronger for STEMI [7]. How might an association of NSTEMI to influenza and of STEMI to weather be explained? A study of biomarkers in MI patients at admission found that patients with STEMI had significantly higher median values of hs-CRP, white blood count, ferritin, and interleukin-6 than did those with NSTEMI, while the only biomarker with higher levels in NSTEMI patients was cluster of differentiation 40 ligand (CD40L) [26, 27]. The CD40L is central to T cell responses during influenza infection, indicates atherosclerotic instability, may mediate interaction of neutrophils and platelets in ACS, and hence suggests a hypothetical link between influenza and NSTEMI [28–30]. The prevalence of plaque rupture and thin-cap fibro-atheroma is higher in STEMI than in NSTEMI, and coronary vasoconstriction upon exposure to cold could alter arterial wall shear stress and lead to plaque rupture [31, 32]. NSTEMI also encompasses type 2 infarctions not dependent on ruptured plaque but on relative ischemia caused by tachycardia or high metabolic state as in fever caused by influenza.

### Strengths and limitations

Strengths of this study include the analysis of data from an entire country over a period greater than seven years. Influenza cases were laboratory confirmed, and MI is registered in SWEDEHEART with a high level of coverage [9]. The study also had a number of limitations. We were unable to pair influenza and MI cases at an individual level and thus unable to draw firm conclusions on biological effects, e.g. that patients suffering from MI had been exposed to influenza, which could lead to ecological fallacy. Meteorological data were based on national averages and not adjusted for local differences in weather. Despite appropriate statistical adjustments, it is possible that unknown confounders may have affected the results of this registry study. For example, information on influenza vaccine status in the studied population was not available. Using only laboratory-confirmed influenza cases implies underreporting, because many individuals with fever, cough, and fatigue do not see a doctor and, if they do, not all are tested for influenza [33]. Finally, although the results observed in our study were statistically significant, the effect estimates were only modest.

## Conclusion

In this nationwide observational study encompassing more than seven years, we found an association of MI with the incidence of influenza cases beyond what could be explained by meteorological factors. Our findings add to accumulating evidence that influenza is an MI trigger.

## Supporting information

S1 Checklist Reporting checklist for cohort study. (DOCX)

S1 Table Results of sensitivity analyses. (DOCX)
