# Supplementary material for: Association of acute myocardial infarction with influenza: A nationwide observational study
Source: PLoS One. 2020 Aug 6;15(8):e0236866. doi: 10.1371/journal.pone.0236866 (PMC7410234; doi:10.1371/journal.pone.0236866)
Supplement: S1 Table — (DOCX) [file pone.0236866.s002.docx]

**S1 Table. Results of sensitivity analyses.**

|  | Cases | | Adjusted IRR (95%CI) | P-value | | |
| --- | --- | --- | --- | --- | --- | --- |
| **Adjusted for weather and month** |  | |  | | |  |
| Total MI | 0-16 | | 0.99 (0.95-1.03) | | 0.670 |  |
|  | 17-163 | | 1.03 (0.99-1.07) | | 0.108 |  |
|  | 164-2382 | | 1.03 (0.99-1.07) | | 0.137 |  |
| Non-ST-Elevation MI | 0-16 | | 1 (0.96-1.05) | | 0.993 |  |
|  | 17-163 | | 1.04 (1-1.08) | | 0.057 |  |
|  | 164-2382 | | 1.05 (1.01-1.09) | | 0.027 |  |
| ST-Elevation MI | 0-16 | | 0.97 (0.93-1.02) | | 0.306 |  |
|  | 17-163 | | 1.01 (0.97-1.05) | | 0.648 |  |
|  | 164-2382 | | 0.99 (0.94-1.04) | | 0.677 |  |
|  |  | |  | |  |  |
| **Unadjusted (Weeks with 0-16 cases as reference period)** |  | | Unadjusted IRR (95%CI) | | |  |
| Total MI | 17-163 | | 1.03 (1-1.06) | | 0.067 |  |
|  | 164-2382 | | 1.04 (1-1.07) | | 0.023 |  |
| Non-ST-Elevation MI | 17-163 | | 1.03 (1-1.07) | | 0.064 |  |
|  | 164-2382 | | 1.05 (1.01-1.09) | | 0.005 |  |
| ST-Elevation MI | 17-163 | | 1.02 (0.98-1.07) | | 0.238 |  |
|  | 164-2382 | | 1.01 (0.97-1.05) | | 0.566 |  |
|  | | | Adjusted IRR (95%CI) | | |  |
| **Adjusted for weather only** | | |  |  |  |  |
| **(Weeks with 0-16 cases as reference period)** | | |  |  |  |  |
| Total MI | | 17-163 | 1.02 (0.99-1.06) | | 0.189 |  |
|  | | 164-2382 | 1.03 (0.99-1.06) | | 0.099 |  |
| Non-ST-Elevation MI | | 17-163 | 1.03 (0.99-1.06) | | 0.148 |  |
|  | | 164-2382 cases | 1.05 (1.01-1.09) | | 0.011 |  |
| ST-Elevation MI | | 17-163 cases | 1.01 (0.97-1.05) | | 0.508 |  |
|  | | 164-2382 cases | 0.99 (0.95-1.03) | | 0.642 |  |
|  | | | Adjusted IRR (95%CI) | | |  |
| **Adjusted for weather and month** | | |  |  |  |  |
| **(Weeks with 0-16 cases as reference period)** | | |  |  |  |  |
| Total MI | | 17-163 | 1.04 (1.01-1.08) | | 0.020 |  |
|  | | 164-2382 | 1.03 (0.99-1.07) | | 0.093 |  |
| Non-ST-Elevation MI | | 17-163 | 1.05 (1-1.09) | | 0.029 |  |
|  | | 164-2382 | 1.05 (1-1.09) | | 0.038 |  |
| ST-Elevation MI | | 17-163 | 1.04 (1-1.09) | | 0.077 |  |
|  | | 164-2382 | 1.01 (0.96-1.06) | | 0.695 |  |
| **Excluding influenza season 2009/2010** | | | 1.08 (1.04-1.11) | | |  |
|  | |  |  |  |  |  |
| Total MI | | 0-16 |  |  |  |  |
|  | | 17-163 | 1.10 (1.06-1.13) | | <0.001 |  |
|  | | 164-2382 | 1.09 (1.05-1.14) | | <0.001 |  |
| Non-ST-Elevation MI | | 0-16 | 1.07 (1.03-1.11) | | <0.001 |  |
|  | | 17-163 | 1.04 (1-1.08) | | <0.001 |  |
|  | | 164-2382 | 1.11 (1.06-1.16) | | <0.001 |  |
| ST-Elevation MI | | 0-16 | 1.09 (1.04-1.13) | | <0.001 |  |
|  | | 17-163 | 1.10 (1.05-1.15) | | <0.001 |  |
|  | | 164-2382 | 1.06 (1.01-1.11) | | 0.01 |  |
